# Evaluation of VectoMax FG application frequency for the control of *Aedes albopictus* and *Culex* species in urban catch basins: evidence from a randomised controlled trial

**DOI:** 10.1186/s13071-025-07169-0

**Published:** 2025-11-27

**Authors:** Tim Kirrmann, Thomas A. Smith, Bianca Modespacher, Pie Müller

**Affiliations:** 1 Swiss Tropical and Public Health Institute, Kreuzstrasse 2, 4123 Allschwil, Switzerland; 2 University of Basel, Petersplatz 1, 4001 Basel, Switzerland

**Keywords:** Mosquito control, *Bacillus thuringiensis* var. *israelensis*, *Lysinibacillus sphaericus*, *Aedes albopictus*, *Culex*, Larval source management, Microbial larvicide

## Abstract

**Background:**

VectoMax FG (Valent BioSciences, Libertyville, IL, USA) is a biological mosquito larvicide, combining *Bacillus thuringiensis* var. *israelensis* and *Lysinibacillus sphaericus*. *Bacillus thuringiensis* var. *israelensis* demonstrates a low propensity for resistance development, whereas *L. sphaericus* exhibits prolonged residual effectiveness in organically polluted aquatic environments. The manufacturer recommends treatments at least every 4 weeks; however, recent evidence suggests that less frequent applications may achieve comparable effectiveness, which is important for reducing operational costs related to larvicide volume and labour as well as reduced environmental exposure.

**Methods:**

To provide data-driven guidance for vector control programmes targeting *Aedes albopictus* in catch basins, we conducted a randomised controlled trial in Basel, Switzerland, from May to October 2024. A total of 180 catch basins, randomly selected from 768 basins in an urban area infested with *Ae. albopictus*, were assigned to treatment intervals of 2, 4, 6, 8 or 10 weeks, alongside untreated controls. Emergence traps were used to capture adult mosquitoes developing from larvae within the basins, allowing comparison of mosquito abundance reductions across treatment frequencies. Generalised additive and linear mixed effects models were applied to quantify the effects of larvicide application frequency, temperature, precipitation and time since treatment on mosquito and non-target dipteran populations.

**Results:**

Suppression of all taxa peaked within 20–30 days post-treatment. Over 50% reductions in mosquito abundance were sustained for up to 10 weeks following treatment, with *Culex* spp. exhibiting persistent suppression exceeding 90% for up to 6 weeks, and *Ae. albopictus* maintaining comparably high levels of suppression for up to 4 weeks.

**Conclusions:**

While *Culex* spp. responded well even at longer intervals, *Ae. albopictus* required more frequent treatment to avoid rebound. Our findings show effective (> 90%) suppression in both *Ae. albopictus* and *Culex* spp. when VectoMax FG was reapplied at 4-week intervals. Increased application frequency not only enhanced overall effectiveness but also reduced variability in mosquito abundance, contributing to more stable vector control.

**Graphical Abstract:**

**Figure:**
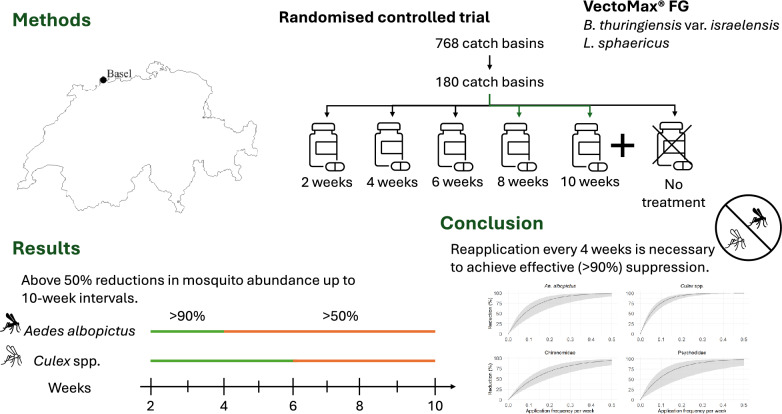

**Supplementary Information:**

The online version contains supplementary material available at 10.1186/s13071-025-07169-0.

## Background

Climate projections indicate a continued expansion of the range suitable for *Ae. albopictus* in Europe, raising significant public health concerns due to its competence as a vector for more than 20 arboviruses, including dengue, chikungunya and Zika [1]. *Aedes albopictus* has already been associated with multiple chikungunya outbreaks in Italy and France [2, 3] and increasing autochthonous dengue outbreaks in Europe. These findings underscore the urgent need for effective mosquito control measures in areas infested with *Ae. albopictus* [4].

Like *Ae. albopictus*, *Culex* spp. have the potential to transmit viruses, including Japanese encephalitis virus (JEV) [5], West Nile virus (WNV) [6] and several other arboviruses [7–10], as well as filarial parasites [11]. The ecological success of *Culex* spp. in urban settings is largely driven by the availability of organic-rich water bodies [12]. Consequently, JEV, WNV, Usutu virus and Sindbis virus have been found in the European *Culex* population in urban environments, indicating the importance of *Culex* spp. control measures [13, 14].

Since its first detection in 2003 [15], *Ae. albopictus* has also become well established in Switzerland, spreading particularly along major transportation corridors [16]. In urban settings, mosquito control strategies typically involve a combination of public awareness campaigns to avoid standing water and larviciding of permanent water bodies such as catch basins [17–19]. In Switzerland, VectoMax FG – a larvicide that combines *Bacillus thuringiensis* var. *israelensis* (*Bti*) and *Lysinibacillus sphaericus* – is routinely used in public mosquito control programmes to treat catch basins [18]. While the manufacturer recommends reapplication every 4 weeks, emerging evidence suggests that longer intervals may still provide effective control and enhance cost-efficiency [20]. Laboratory studies have shown that formulations combining *Bti* and *L. sphaericus*, such as VectoMax FG, can achieve greater than 80% reduction in both *Culex quinquefasciatus* and *Aedes aegypti* populations for up to 8 weeks. This extended efficacy is largely attributed to the synergistic action of the Cyt1Aa toxin in *Bti*, which facilitates the entry of binary toxins (Bin) into the epithelial cells of larvae that are resistant or lack Bin receptors [21].

To evaluate the effect of VectoMax FG application frequency on reducing the number of adult mosquitoes, we conducted a randomised controlled trial in the St. Johann district in Basel, Switzerland, during the 2024 mosquito season. Catch basins were treated at different frequencies, ranging from 2- to 10-week intervals, and their effects on mosquito abundance were measured via adult emergence traps placed in the catch basins.

## Methods

### Study area

The St. Johann district, located in the north-west of the city of Basel, Switzerland, was selected because of its well-documented high prevalence of *Ae. albopictus* [22]. Basel is highly urbanised with dense residential infrastructure and many underground catch basins that provide ideal conditions for container-breeding mosquitoes.

### Weather data

The daily mean air temperature and total precipitation were obtained from the nearest MeteoSwiss stations – Basel-St. Johann (temperature at 2 m above ground) and Basel-Binningen (precipitation).

### Treatment procedure

**Fig. 1:**
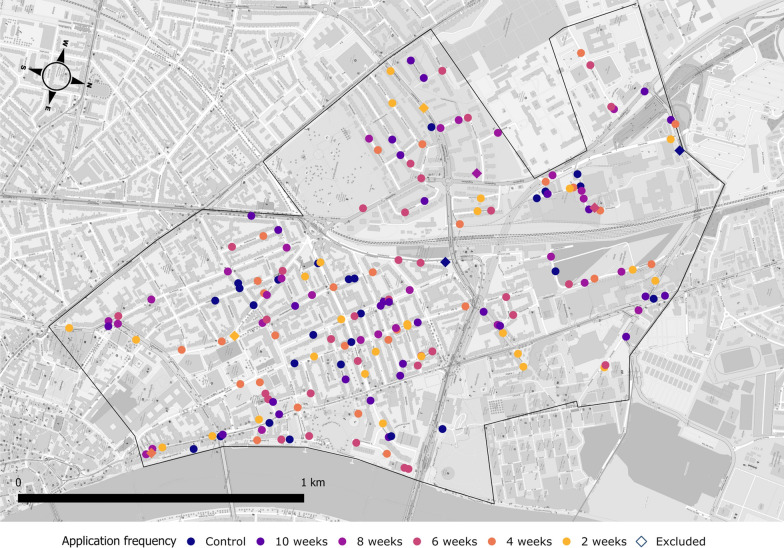
Map of catch basins included in the study in the St. Johann district, Basel, Switzerland. Each point represents a catch basin, and the colour indicates the respective larvicide application frequency (treatment). From a list of 768 catch basins located at St. Johan, 30 were randomly assigned to each of the 6 treatments (i.e. a total of 180 catch basins). The black line indicates the district border of St. Johann, Basel. The map was created in QGIS version 3.43.13, incorporating a base layer from OpenStreetMap ( ^©^OpenStreetMap contributors)

From a list of 768 catch basins in St. Johann, provided by the local administration, 30 were randomly assigned to each of five larvicide application frequencies – 2, 4, 6, 8 or 10 weeks – or to a negative control group that received no treatment (Fig. 1). Each treatment consisted of VectoMax FG (Valent BioSciences, Libertyville, IL, USA), a formulation combining *Bti* (strain AM65-52) and *L. sphaericus* (strain ABTS 1743), applied at a dose of 10 g per catch basin in accordance with the label. A pre-measured 10 g cup was used to dispense the granules into each catch basin through a funnel to minimise spillage. Treatments commenced on 29 April and continued until 24 October 2024. During that period, catch basins treated at frequencies of 2, 4, 6, 8 and 10 weeks received 13, 7, 5, 4 and 3 treatments, respectively.

### Mosquito collection procedure

**Fig. 2:**
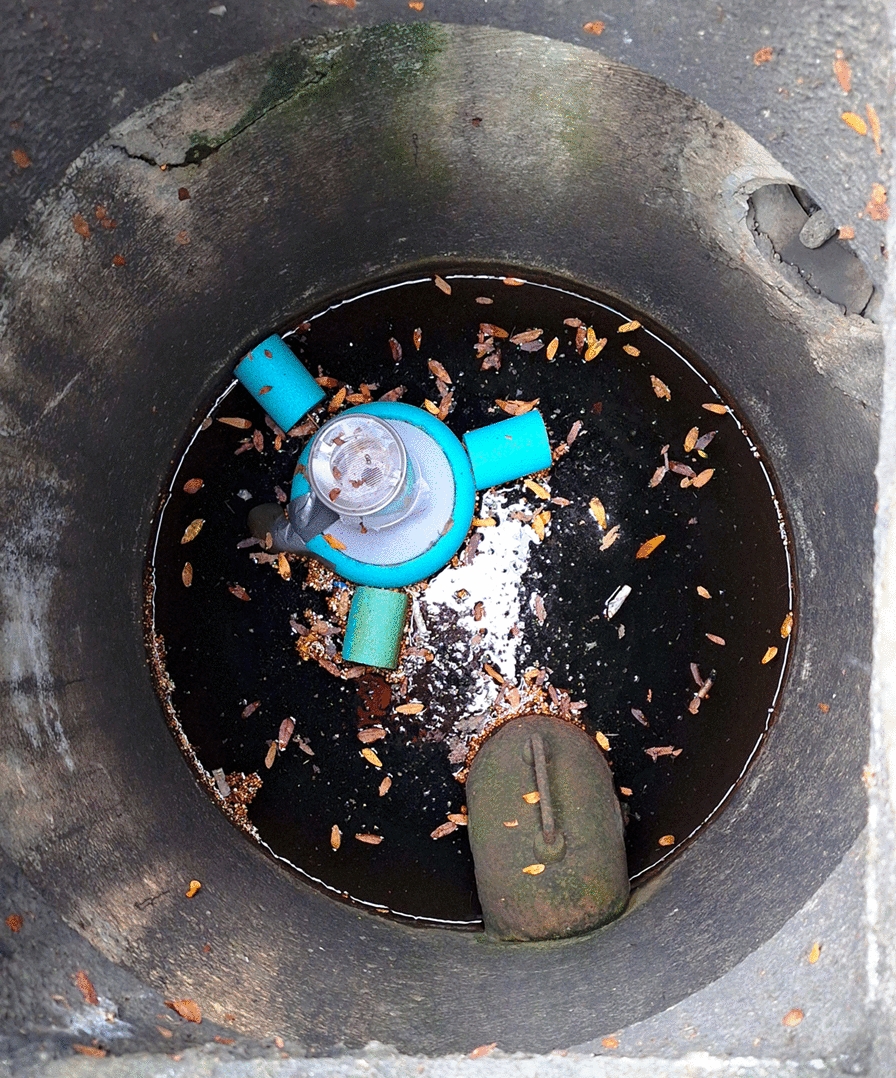
Adult emergence trap installed in a catch basin to assess the effectiveness and optimal reapplication interval of larvicide treatments. The trap captures emerging adult mosquitoes, providing direct evidence of larval survival following treatment

The emergence of adult mosquitoes, and other dipterans, from the selected catch basins was monitored weekly via custom-built adult emergence traps on the basis of the design of Ravasi et al. [20]. Adult emergence traps were employed to determine the actual number of adult mosquitoes emerging after VectoMax FG treatment. In contrast to larval sampling methods commonly used in similar studies, it remains uncertain whether the larvae collected in catch basins would have successfully developed into adults under natural conditions. Each trap consisted of a white plastic funnel with a diameter of 16.1 cm. Low-density polyethylene (LDPE) foam was attached around the base to allow the traps to float on the water, resulting in a total diameter of 20.5 cm. The openings of the funnels were 1.7 cm wide and led directly into collection cups, measuring 9.1 cm in diameter and 10.1 cm in height. Each cup had a 5.1-cm opening at the base covered with a fine mesh net. Fine mesh nets with small openings were also attached to the tops of the cups, allowing mosquitoes to enter through the funnel while preventing their escape during cup removal and replacement. Traps were placed inside the catch basins, floating on the water surface to capture emerging adult dipterans. To ensure stability, three 10-cm LDPE foam pieces were attached to the base of each trap (Fig. 2). Traps were removed and replaced on a weekly basis, during which their functionality – including net integrity and placement – was checked, alongside catch basin conditions such as water level and the presence of leaves or other debris.

Each cup was labelled with the corresponding catch basin ID and the week of collection to ensure traceability of the samples. Mosquitoes were brought to the laboratory and frozen at −18 °C for at least 2 h. The samples were then transferred into 1.5-ml Eppendorf tubes in pools of up to 10 individuals and stored at −18 °C until later identification.

### Mosquito identification

At the conclusion of the field season, all frozen insects were identified morphologically to major taxonomic groups under a stereo microscope, using the ‘Reverse identification key for mosquito species’ by the European Centre for Disease Prevention and Control and the ‘Key to Diptera families – adults’ [23–25]. The taxonomic groups included *Culex* spp., Chironomidae, Psychodidae and *Aedes* spp. Specimens belonging to the genus *Aedes* were further identified to the species level.

### Data analysis

The data were first recorded on paper forms and then transferred to an Open Data Kit database [26]. Subsequent data analysis was performed in R version 4.51 [27], including data on the four taxonomic groups *Ae. albopictus*, *Culex* spp., Chironomidae and Psychodidae.

Abundance was summarised as the arithmetic mean number of specimens per taxonomic group and per adult emergence trap per week, with 95% confidence intervals (CIs) estimated using the R package ‘boot’ [28], on the basis of 1000 bootstrap samples. This approach allowed for the estimation of 95% CIs without requiring preliminary assumptions regarding the distribution, thereby providing CIs that more reliably reflect the observed data.

To evaluate the effects of larvicide application frequency, temperature and precipitation on mosquito abundance, generalised linear mixed models (GLMMs) and generalised additive mixed models (GAMMs) were developed, using the R packages ‘glmmTMB’ [29] and ‘mgcv’ [30], respectively. For the models, the temperature was centred around the mean of 19.7 °C. To account for potential delays between weather conditions and changes in mosquito abundance several lag periods (0–21 days) for both temperature and precipitation were assessed. On the basis of biological plausibility and preliminary model performance, only a 1-week lag for precipitation was selected for the final models. Owing to overdispersion in the mosquito count data, models were fitted via a negative binomial error distribution, and separate models were constructed for each taxonomic group to account for taxon-specific responses.

First, a GLMM with categorical application frequency was used to quantify the associations between application frequency, temperature and lagged precipitation while accounting for temporal and spatial correlation through random intercepts (Eq. 1). Table 1 explains the different variables and model terms used in the statistical models described above.

1 $$\begin{array}{*{20}c} {\nu_{i} \in 0.1,0.125,0.167,0.2,0.5} \\ {log\left( {E\left[ {Y_{i} } \right]} \right) = \beta_{0} + \sum {_{j = 1}^{5} \beta_{j} } 1\left( {\nu_{i} = j} \right) + \beta_{6} \tau_{i} + \beta_{7} \rho + \mu_{1} \left[ i \right] + \mu_{2} \left[ i \right]} \\ \end{array}$$

**Table 1:** Variables and model terms used in the linear models

Variable	Description	Units
*Y*_1_	Observed mean mosquito count at trap *i*	$\overline{x }$
*E*[*Y*_*i*_],${\eta }_{i}$	Expected mosquito	Mean count (per trap)
*υ*_*i*_	Frequency of larvicide application at trap *i*	–
1(*υy*_*i*_ = *j*)	Indicator function for application frequency level *j*	$\in 0.1, 0.125, 0.167, 0.2, 0.5$
*τ*_*i*_	Mean temperature at trap *i* during sampling period	°C
*ρi*	Precipitation 1 week after sampling at trap *i*	mm
*t*_*i*_	Time since larvicide was last applied at trap *i*	Days
*μ*_1_[*i*]	Random intercept for trap location	1–180
*μ*_2_[*i*]	Random intercept for sampling day	Calendar days
*b*_1_[*i*]	Random effect for trap (in GAMM context)	1–180
*b*_2_[*i*]	Random effect for day (in GAMM context)	Calendar days
*f*(·)	Smooth function (thin plate spline)	–
*β*_0_	Intercept (fixed effect)	Mean count (per trap)
*β*_*j*_	Coefficients for model predictors	Mean count (per trap)
SE_*β*_1_	Standard error of frequency coefficient	–
*x*	Input frequency sequence used for predictions	seq(0, 0.5, by = 0.001)
R	Predicted mosquito reduction = 100(1 − exp(*β*_1_*x*))	%

Next, the observed mean trap counts with 95% CIs were plotted to illustrate the relationship between mosquito abundance and time since the most recent treatment. To better understand these trends, model-based estimates of mean trap counts generated from GAMMs were overlaid. To establish a baseline for comparison, initial mosquito abundance was estimated by setting the ‘time since treatment’ to zero in untreated control catch basins. Although equivalent GLMMs using the same predictors yielded lower Akaike information criterion (AIC) values, diagnostic checks revealed consistent nonlinear patterns in abundance over time across all taxa. Therefore, GAMMs were selected for their greater flexibility in capturing these dynamics (Eq. 2).

2 $$\begin{array}{*{20}c} {log\left( {\eta_{i} } \right) = f\left( {t_{i} } \right) + \beta_{2} \tau_{i} + \beta_{3} \rho_{i} + b_{1} \left[ i \right] + b_{2} \left[ i \right]} \\ \end{array}$$

Finally, a GLMM including application frequency as a continuous variable was used to estimate the dose–response relationship between application frequency and the number of emerging adult insects. The predicted insect reduction rates were derived across application frequencies and taxonomic groups (Eq. 3).

3 $$\begin{array}{*{20}c} {\begin{array}{*{20}c} {log\left( {E\left[ {Y_{i} } \right]} \right) = \beta_{0} + \beta_{1} \upsilon_{i} + \beta_{2} \tau_{i} + \beta_{3} \rho + \mu_{1} \left[ i \right] + \mu_{2} \left[ i \right]} \\ \end{array} } \\ \end{array}$$

Although the inclusion of precipitation and temperature did not consistently improve model fit, as indicated by the AIC, across all taxonomic groups, these variables were retained to ensure comparability between groups.

## Results

During the sampling period, seven catch basins were excluded owing to the absence of water accumulation (4), construction work obstructing access (1), flooding of a residential area due to blockage after heavy rain (1) and incidental larvicidal treatment by a third party (1) (Fig. 1). A total of two, two, one, one, one and zero catch basins were excluded for the control and the 2-, 4-, 6-, 8- and 10-week intervals, respectively. The remaining 173 catch basins were monitored weekly. No sampling was executed from 23 to 30 September 2024. A total of 2076 trap observations were recorded with 12.6% of the data containing missing values, primarily owing to trap displacement caused by heavy rainfall.

More than half of the collection cups (58.8%) were empty, whereas the remaining ones contained one or more individuals, resulting in a total of 4936 collected dipterans. Owing to desiccation or physical damage, 3.4% of specimens could not be reliably identified, leaving 4768 individuals for taxonomic classification. Most of the specimens caught in the traps were *Culex* spp. (53.2%), followed by Chironomidae spp. (31.1%), Psychodidae (6.4%) and *Ae. albopictus*. (5.8%). A randomly selected subsample of 81 *Culex* spp. was identified as entirely *Culex pipiens*, except for one specimen that could only be identified to the *Culex* genus level, supporting the assumption that most *Culex* specimens were likely *Cx. pipiens*. All Psychodidae were members of the subfamily Psychodinae.

### Effect of application frequency on the abundance of mosquitoes and non-target species

**Fig. 3:**
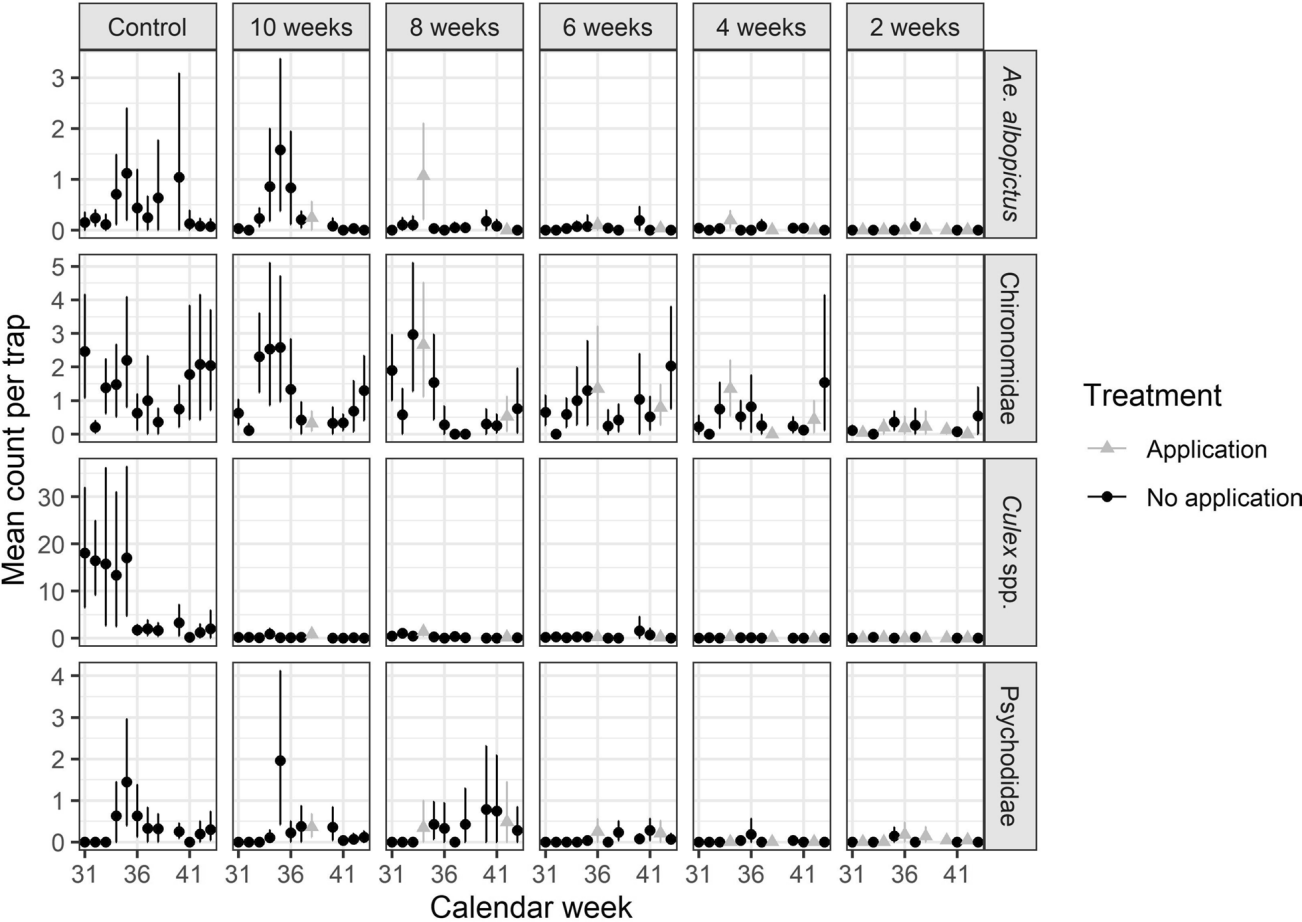
Abundance of mosquitoes and non-target insects in untreated and treated catch basins. Each point represents the arithmetic mean count per trap and ISO calendar week for the four taxonomic groups. The whiskers represent the 95% confidence intervals around the mean count. No sampling was executed in week 39

Analysis of the untreated control catch basins revealed clear seasonal patterns in dipteran abundance, with *Culex* spp. showing the highest activity (7.72 individuals/trap), followed by Chironomidae (1.36), *Ae. albopictus* (0.41) and Psychodidae (0.34). The peak activity for all taxonomic groups occurred around (ISO) calendar week 35 (Fig. 3). Intriguingly, *Culex* spp. exhibited a sharp peak followed by a rapid decline, whereas *Ae. albopictus* maintained low but stable numbers throughout the season, exceeding one individual only during calendar weeks 35 and 40 (Fig. 3).

Larvicide treatments with VectoMax FG substantially suppressed adult emergence compared with the untreated catch basins, with reductions most pronounced within 1 to 2 weeks post-application (Fig. 3). For *Ae. albopictus*, application frequencies of 2 to 6 weeks maintained low abundance (0.01–0.05 mosquitoes/trap), whereas frequencies of 8 and 10 weeks were less effective, particularly the 10-week interval, which closely resembled control levels. The model results (Eq. 1) indicated statistically significant suppression at application intervals of 6 weeks or shorter, with no significant associations observed for temperature or precipitation (Table 2).

**Table 2:** Effects of VectoMax FG application frequencies on the abundance of *Aedes albopictus*, *Culex* spp., Chironomidae and Psychodidae in catch basins

Taxon	Model predictor	Coefficient	95% CI	*P*-value
*Aedes albopictus*	Frequency (10 weeks)	1.07	0.31–3.68	n.s.
	Frequency (8 weeks)	0.47	0.13–1.72	n.s.
	Frequency (6 weeks)	0.16	0.04–0.64	0.010
	Frequency (4 weeks)	0.11	0.03–0.49	0.004
	Frequency (2 weeks)	0.02	0.00–0.17	< 0.001
	Temperature (°C)	1.05	0.98–1.14	n.s.
	Precipitation (mm)^1^	0.93	0.85–1.01	n.s.
*Culex* spp.	Frequency (10 weeks)	0.02	0.01–0.08	< 0.001
	Frequency (8 weeks)	0.06	0.02–0.18	< 0.001
	Frequency (6 weeks)	0.01	0.00–0.04	< 0.001
	Frequency (4 weeks)	0.00	0.00–0.01	< 0.001
	Frequency (2 weeks)	0.00	0.00–0.01	< 0.001
	Temperature (°C)	1.20	1.10–1.30	< 0.001
	Precipitation (mm)^1^	0.92	0.85–0.99	0.024
Chironomidae	Frequency (10 weeks)	0.59	0.21–1.63	n.s.
	Frequency (8 weeks)	0.39	0.14–1.12	n.s.
	Frequency (6 weeks)	0.37	0.13–1.04	n.s.
	Frequency (4 weeks)	0.19	0.07–0.57	0.002
	Frequency (2 weeks)	0.06	0.02–0.19	< 0.001
	Temperature (°C)	1.06	1.00–1.12	0.039
	Precipitation (mm)^1^	0.98	0.95–1.02	n.s.
Psychodidae	Frequency (10 weeks)	0.50	0.08–3.01	n.s.
	Frequency (8 weeks)	0.17	0.02–1.20	n.s.
	Frequency (6 weeks)	0.16	0.03–1.07	n.s.
	Frequency (4 weeks)	0.02	0.00–0.19	< 0.001
	Frequency (2 weeks)	0.03	0.00–0.25	0.001
	Temperature (°C)	0.85	0.76–0.96	0.01
	Precipitation (mm)^1^	0.96	0.92–1.01	n.s.

^1^Precipitation with a lag of 1 week. 95% CI, 95% confidence interval. Estimates and 95% CIs are based on the GLMM in Eq. 1

Compared with those of the control, *Culex* spp. trap counts were consistently lower across all application frequencies (Fig. 3). The GLMM results from Eq. 1 confirmed a significant negative association between all treatment intervals and *Culex* spp. abundance (Table 2). The effect of VectoMax FG was also more pronounced on *Culex* spp. than on *Ae. albopictus*, indicating greater sensitivity to the larvicide. Additionally, temperature was positively associated with *Culex* spp. count, whereas precipitation was negatively associated with *Culex* spp. count (Table 2). In chironomids and psychodids, the model results (Eq. 1) indicate a significant decline in abundance at intervals of 4 weeks or shorter. Temperature had a statistically significant positive effect on chironomid abundance but a statistically significant negative effect on psychodids, whereas precipitation was not significantly associated with either group (Table 2).

### Residual effect on adult insect abundance

**Fig. 4:**
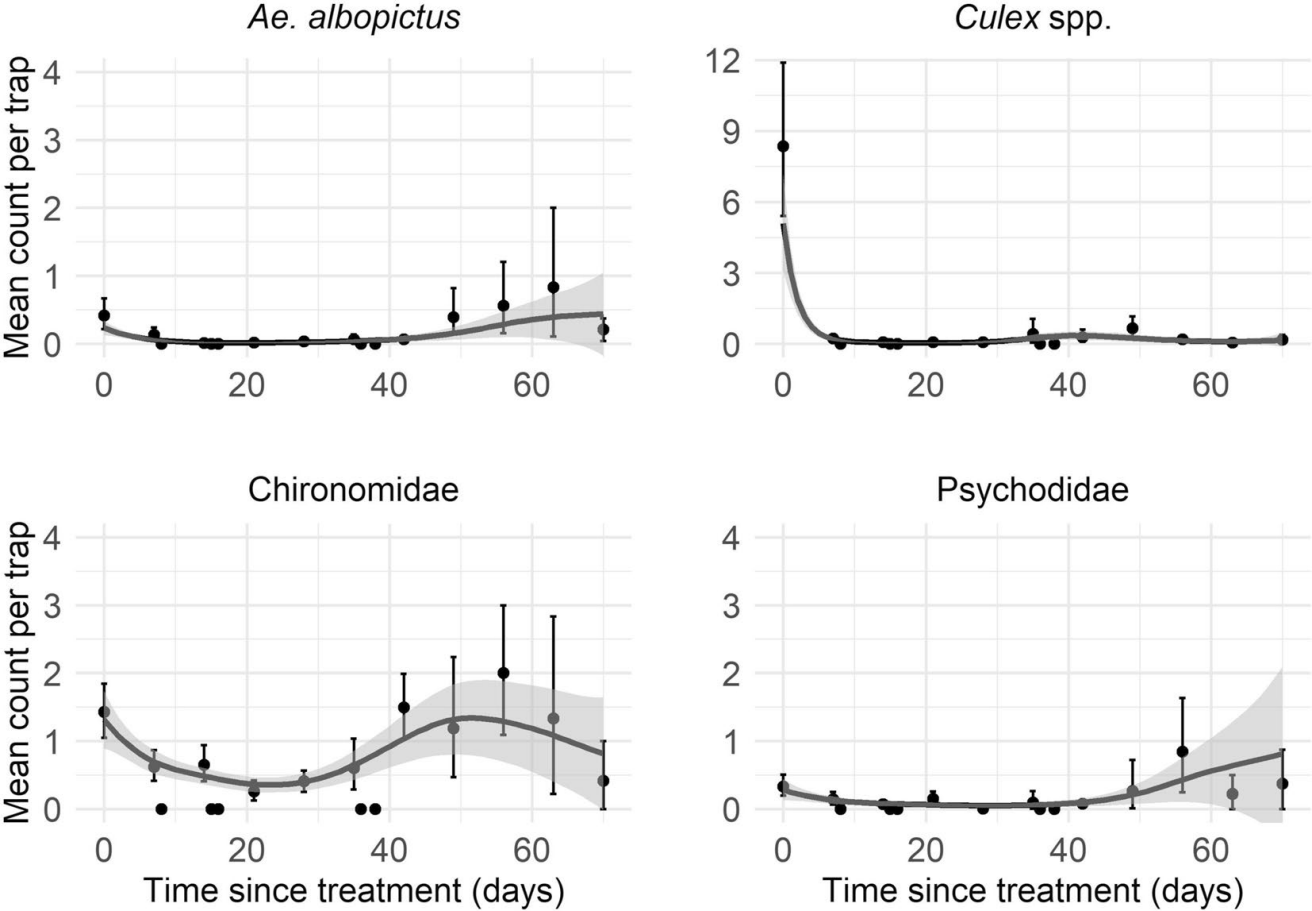
Residual effects of VectoMax FG in catch basins against mosquitoes and non-target insects. Each point represents the estimated mean number of adult insects per trap and days post treatment, with whiskers representing the 95% confidence interval around the means. The lines show the predicted abundance as a function of time post treatment estimated from GAMMs (Eq. 2). The shaded areas show the 95% confidence ranges around the predicted lines

The model results (Eq. 2) on residual effects revealed significant non-linear effects of time since treatment across all the taxa (Fig. 4). Incorporating a random intercept for trap ID substantially improved model fit by accounting for consistent differences among traps, indicating that traps with higher mosquito counts in 1 week tended to have higher counts in subsequent weeks. Additionally, calendar time (days) was a good predictor of *Ae. albopictus*, *Culex* spp. and Chironomidae abundance, whereas no significant temporal trend was observed for Psychodidae abundance. Among the environmental covariates, 1-week-lagged precipitation and a 1 °C increase above the mean temperature significantly influenced Chironomidae abundance, with temperature showing a positive association and precipitation showing a negative association (Additional file 1: Table S1). In contrast, only precipitation had a statistically significant negative effect on *Ae. albopictus* and *Culex* spp. abundance, whereas no significant associations were observed for Psychodidae. Suppression of both target and non-target taxa was most pronounced shortly after treatment, with predicted abundance reductions peaking during the first 19 days for *Culex* spp., 20 days for *Ae. albopictus*, 23 days for Chironomidae and 29 days for Psychodidae. *Culex* spp. showed sustained suppression of ≥ 80% throughout the study, whereas *Ae. albopictus* remained above 80% suppression until day 36 and retained an effectiveness of ≥ 50% up to day 46. Among the non-target taxa, Chironomidae experienced ≥ 50% reductions lasting through day 35, whereas Psychodidae showed sharper and more prolonged suppression, with reductions exceeding 80% up to day 34 and remaining ≥ 50% until day 45.

### Dose–response effects of application frequency on adult insect abundance

**Fig. 5:**
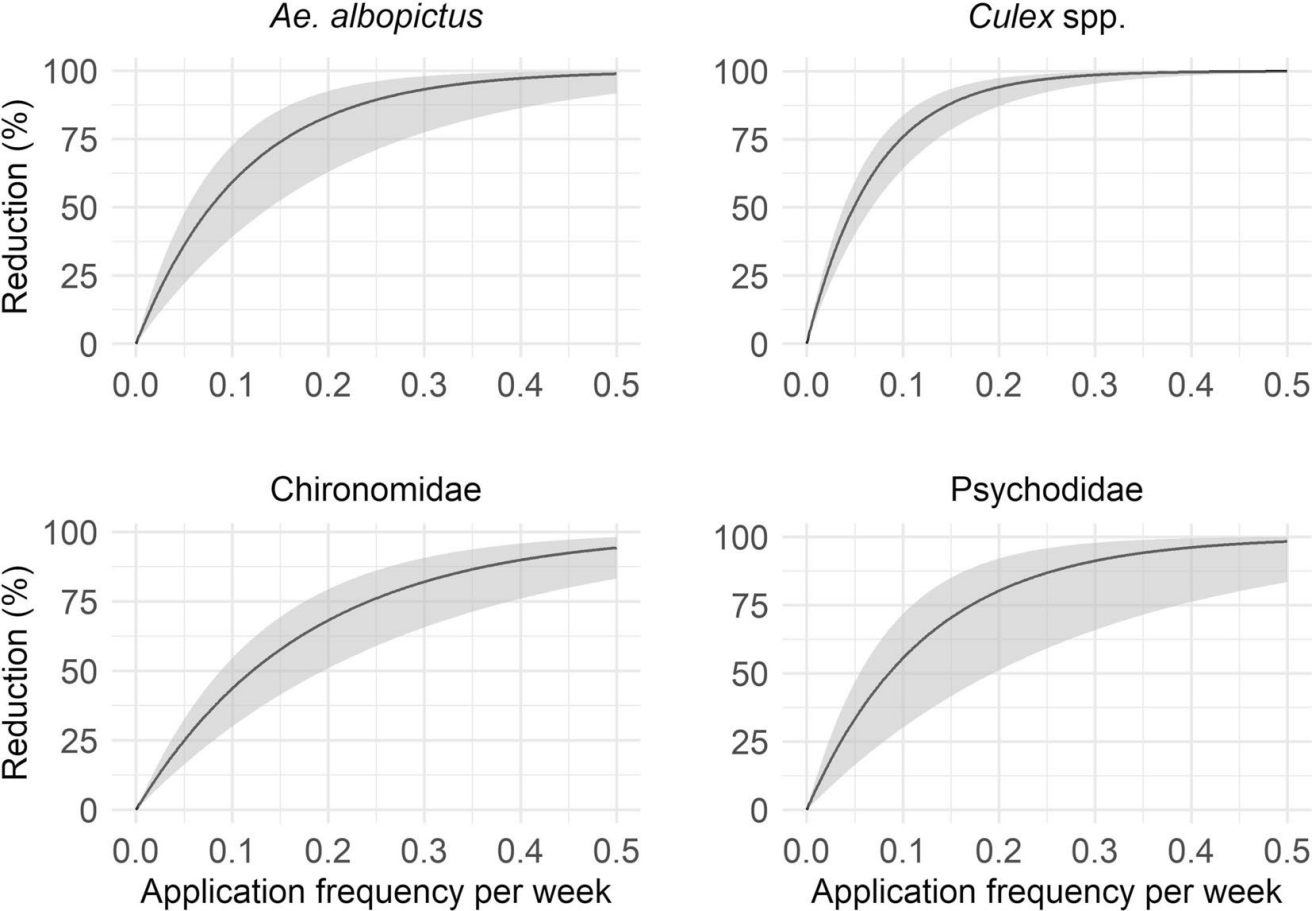
Percent reduction in mosquito and non-target insect abundance as compared with that in the control group as a function of VectoMax FG treatment application frequency. The predictions are based on the fitted GLMM (Eq. 3), allowing exploration across intermediate treatment intervals. Specific values for treatment intervals from 2 to 10 weeks are given in Additional file 1 (Table S2)

In the continuous dose–response model (Eq. 3), the *Ae. albopictus* abundance was significantly influenced solely by application frequency, which was strongly negatively associated with the number of *Ae. albopictus* (Fig. 5, Additional file 1: Table S2). Neither temperature nor lagged precipitation had a statistically significant effect. Model predictions indicate that reductions in *Ae. albopictus* abundance increased with more frequent applications, with a 73.9% reduction at the 8-week interval, which is currently implemented in several urban areas in Switzerland (pers. comm. Swiss Mosquito Network). According to the model prediction, suppression exceeds 80% at the 5-week interval, surpasses 90% with treatments every 4 weeks, and approaches near-complete control (99%) with biweekly applications. Additionally, variability in predicted abundance declined sharply with increasing treatment frequency, as reflected by narrowing 95% CIs, suggesting a reduced risk of population resurgence. Marginal gains in reduction increased steadily up to the 4-week interval, peaking at 5.7%, before subsequently declining.

In contrast, *Culex* spp. abundance was significantly affected by all three predictors. The application frequency has a strong negative effect; temperature is positively associated – each 1 °C increase above the mean (19.7 °C) significantly elevates counts – and precipitation lag exerts a smaller but significant negative effect (Fig. 5, Additional file 1: Table S3). The predicted reductions in *Culex* spp. exceeds 80% – even at the 8 week application interval – and reaches over a 90% reduction with an application frequency every 6 weeks, and near-complete suppression at 2- to 3-week intervals. Like in *Ae*. *albopictus*, variability in abundance across catch basins declines as application frequency increases, although re-emergence fluctuations are less pronounced. Marginal gains peak at the 7-week interval (3.8%) and decrease rapidly at shorter application frequencies (Fig. 5, Additional file 1: Table S3).

For the non-target taxonomic groups, application frequency is also a significant predictor. Temperature has contrasting effects, with a positive relationship observed for Chironomidae abundance and a negative association for Psychodidae abundance. Precipitation lag was not a significant predictor for either group (Additional file 1: Table S3). The predicted reductions in non-target abundance increased with higher treatment frequency, with the abundance of Chironomidae being reduced by 43.6% at 10 week intervals and up to 94.3% under biweekly treatments. The degree of Psychodidae reduction ranged from 55.5% (10-week interval) to 98.3% (2-week interval). Marginal gains differed between taxa: Chironomidae gains increased steadily with shorter intervals, reaching a maximum of 9.2%, whereas Psychodidae gains peaked at a 6-week interval (6.6%) before diminishing at higher frequencies (Fig. 5, Additional file 1: Table S3).

## Discussion

This study demonstrated that the frequency of VectoMax FG application is a critical factor for effectively controlling *Ae. albopictus* and *Culex* spp. in urban catch basins. While both taxa were significantly suppressed, *Culex* spp. responded consistently well across all the tested frequencies, indicating increased sensitivity to the larvicide. Application intervals longer than 4 weeks led to reduced effectiveness and increased variability in mosquito counts, particularly for *Ae. albopictus*. Across both taxa, more frequent treatments resulted in narrower confidence intervals, indicating greater stability in population control. These findings support the manufacturer’s recommended 4-week interval and caution against extending application intervals beyond 4 weeks, especially in areas where *Ae. albopictus* is a primary concern.

This study has several limitations to consider. The adult mosquito emergence traps used lacked protective covers, which may have allowed rainwater to flush out mosquitoes, potentially lowering catch numbers and complicating the assessment of precipitation effects. While the measurement error of these traps has not been fully quantified, a comparison of their seasonal trends with those of previous years revealed consistent patterns, supporting their effectiveness for temporal monitoring [31, 32]. Microclimatic conditions within catch basins – such as temperature, humidity, and water retention – were not continuously monitored, limiting our understanding of how basin-specific environmental variability may influence larvicide persistence or mosquito emergence. This is particularly relevant for *Ae. albopictus*, which remained active until late October, suggesting that thermal buffering within catch basins may have extended its breeding season [33]. Furthermore, although the biological response to larvicide application frequency is inherently non-linear, we adopted a linear representation to provide a simplified and practical framework for operational guidance. This trade-off balances model complexity with clarity, offering control programmes a more straightforward decision-making tool. Finally, as this study was conducted within a single urban region, caution should be exercised in generalising the findings to areas with different climatic, infrastructural or ecological characteristics.

*Bacillus thuringiensis* var. *israelensis* and *L. sphaericus* are key components of environmentally sustainable vector control programmes, being valued for their high specificity towards mosquito larvae and minimal toxicity to vertebrates and most non-target organisms. These microbial larvicides act through parasporal crystal protoxins that, upon ingestion, are solubilised and activated in the alkaline midgut of mosquito larvae. The activated toxins bind to specific receptors on midgut epithelial cells, leading to pore formation, cell lysis and larval death. *Bacillus thuringiensis* var. *israelensis* is known for its rapid action but limited environmental persistence, typically requiring reapplication within 2–4 weeks [34]. In contrast, *L. sphaericus* exhibits longer-lasting control, owing to its ability to reproduce within the cadavers of affected insect larvae, thereby sustaining its larvicidal activity through recycling. When used in combination, *Bti* and *L. sphaericus* are intended to provide both immediate and prolonged mosquito control [35]. Indeed, the dual-action formulation of VectoMax FG – which combines *Bti* for rapid knockdown with *L. sphaericus* for residual activity – appears to provide a synergistic benefit [36]. Nonetheless, the progressive decline in efficacy beyond 3 to 5 weeks post-treatment suggests that the residual component alone is insufficient for sustained suppression of species such as *Ae. albopictus*, underscoring the need for timely reapplications.

The results highlight the importance of tailoring larvicide application intervals to species-specific biology. The higher sensitivity of *Culex* spp. may reflect ecological and physiological traits, such as slower feeding rates, habitat preferences and increased susceptibility to *L. sphaericus* components of the larvicide [37, 38]. Previous studies support these patterns; for example, competitive interactions under limited food conditions have shown that *Ae. aegypti* can outcompete *Cx. quinquefasciatus*, potentially explaining some of the observed differences [39].

Comparisons with previous studies confirm the general pattern of residual activity following VectoMax FG application but also highlight notable variability across different ecological settings. For example, a semi-field trial conducted in Brazil demonstrated the suppression of *Ae. albopictus* for up to 8 weeks and *Culex* spp. for 9 weeks using a similar larvicide formulation [21]. Similarly, a field study in Ticino, Switzerland, reported reductions of approximately 60% in *Ae. albopictus* and up to 85% in *Culex* spp. within 5–10 weeks post-application [20]. These findings are broadly consistent with our results; however, our data suggest a need for more frequent reapplications to sustain high control levels, particularly for *Ae. albopictus*, potentially reflecting local environmental factors.

One study documented the efficacy of VectoMax FG in challenging field conditions, including vegetated environments with high organic content, where residual effects against *Culex* spp. persisted for up to 36 days [35]. Laboratory studies under controlled conditions have reported even longer durations of efficacy, although often at application rates substantially exceeding field recommendations. For example, one study using 57.7 g/m^2^ VectoMax FG in catch basin analogues reported complete suppression for up to 1 year [40], whereas other laboratory experiments achieved > 80% control of *Ae. aegypti* for over 23 weeks with elevated *Bti* concentrations [41]. These extended durations are unlikely to be replicated under field conditions because of the environmental degradation of active ingredients. As seen in the present study, susceptibility patterns among target species further complicate efficacy outcomes. *Culex quinquefasciatus* and *Anopheles gambiae* exhibit comparable reduction rates during the first 9 days following treatment with *Bti* or *L. sphaericus* [42]. In contrast, *Ae. aegypti* has notably lower susceptibility to *L. sphaericus*, with minimal reductions observed at practical dosages, whereas *Ae. albopictus* appears moderately susceptible [43].

In addition to efficacy, ecological safety remains a cornerstone of integrated mosquito management strategies. Despite their generally high specificity, larvicide treatments can have unintended ecological effects on non-target dipteran taxa such as chironomids and psychodids. These taxa play critical roles in the decomposition of organic matter and nutrient cycling in aquatic ecosystems, particularly in polluted or eutrophic habitats such as urban catch basins. Disruption of these communities may have cascading effects on higher trophic levels and alter ecosystem functioning [34, 44, 45]. The Ticino study reported a 50% reduction in chironomid populations 7 weeks after *Bti* application [20], a pattern found in our findings and in others, such as a semi-field trial in Germany, which reported chironomid reductions ranging from 39% to 68% [44]. While these reductions indicate some non-target effects, their magnitude remains substantially lower than that observed for mosquito larvae, supporting the selective action of bacterial larvicides. Furthermore, a field study in Cameroon found no significant changes in zooplankton or macroinvertebrate diversity or abundance following VectoMax FG application, reinforcing its ecological compatibility when used according to guidelines [46]. Similarly, other studies have reported minimal effects of *Bti* on aquatic nutrient dynamics and no measurable impacts on riparian spider populations, suggesting limited disruption to broader ecosystem functions [47].

Our findings reinforce the importance of following the label instructions provided by the manufacturer when applying VectoMax FG. Balancing effective mosquito suppression with ecological considerations – particularly with respect to non-target impacts – requires careful calibration of treatment schedules to optimise both vector control and environmental stewardship.

Future studies should aim to better understand the influence of environmental – particularly rainfall – on mosquito catch data by employing traps equipped with rain shields and by directly measuring water levels within catch basins. In addition, incorporating continuous microclimate monitoring, including temperature and humidity sensors, would offer valuable insights into site-specific conditions that may influence larvicide persistence and mosquito emergence. This is especially relevant for *Ae. albopictus*, which remained active through late October in our study area, indicating a potential shift in seasonal emergence patterns likely driven by thermal buffering within urban catch basins. A more detailed understanding of these microclimatic dynamics could inform the timing and frequency of larvicide applications and help ensure more reliable seasonal coverage.

## Conclusions

Our findings indicate that reapplication of VectoMax FG at 4-week intervals is necessary to achieve effective (> 90%) suppression of both *Ae. albopictus* and *Culex* spp. While *Culex* spp. showed robust sensitivity even at extended intervals, *Ae. albopictus* required more frequent treatments to prevent population rebound. The application frequency influenced not only the suppression level but also the variability in mosquito abundance, contributing to more stable vector control.

## Supplementary Information

Additional file 1.

Additional file 2.

## Data Availability

The dataset supporting the conclusions of this article is included in Additional file 2.

## Abbreviations

*Ae.*: *Aedes*
*Bti*: *Bacillus thuringiensis* var. *israelensis*
*Cx.*: *Culex*
FG: Fine granule
LDPE: Low-density polyethylene
Bin: Binary toxin
*CI*: Confidence interval
GAMM: Generalised additive mixed effects model
GLMM: Generalised linear mixed effects model
JEV: Japanese encephalitis virus
*L*: *Lysinibacillus*
*WNV*: *West Nile virus*
