# Supplementary material for: Evaluation of VectoMax FG application frequency for the control of Aedes albopictus and Culex species in urban catch basins: evidence from a randomised controlled trial
Source: Parasit Vectors. 2025 Nov 27;18:511. doi: 10.1186/s13071-025-07169-0 (PMC12752035; doi:10.1186/s13071-025-07169-0)
Supplement: Supplementary file 1 — Additional file 1. [file 13071_2025_7169_MOESM1_ESM.pdf]

Additional file 1

Evaluation of VectoMax FG application frequency for the control of *Aedes albopictus* and *Culex* species in urban catch basins: evidence from a randomised controlled trial

Tim Kirrmann[1,2], Thomas A. Smith[1,2], Bianca Modespacher[1,2], Pie Müller[1,2,*]

[1] Swiss Tropical and Public Health Institute, Kreuzstrasse 2, 4123 Allschwil, Switzerland
[2] University of Basel, Petersplatz 1, 4001 Basel, Switzerland

*Correspondence: pie.mueller@swisstph.ch

Table S1 GAMM model estimates for the effects of VectoMax FG, temperature and precipitation on the abundance in catch basins of mosquitoes and non-target insects

Aedes albopictus

**Model predictors**	**Coefficient**	**SE**	***P*-value**	**95% CI**
Temperature[1]	0.917	0.045	0.052	0.841 - 1.001
Precipitation (1-week lag)	0.855	0.052	0.003	0.772 - 0.947
**Smooth terms**	**edf**	***χ2***	***P*-value**	
Days since treatment	4.654	72.770	<0.001	
Trap ID (random effect)	0.958	22.020	<0.001	
Calendar days (random effect)	0.915	10.110	0.001	

***Culex* spp.**

**Model predictors**	**Coefficient**	**SE**	***P*-value**	**95% CI**
Temperature[1]	1.050	0.037	0.185	0.977 - 1.129
Precipitation (1-week lag)	0.915	0.030	0.003	0.862 - 0.970
**Smooth terms**	**edf**	***χ2***	***P*-value**	
Days since treatment	6.341	270.115	<0.001	
Trap ID (random effect)	0.958	21.671	0.000	
Calendar days (random effect)	0.829	4.712	0.017	

Chironimidae

**Model predictors**	**Coefficient**	**SE**	***P*-value**	**95% CI**
Temperature[1]	1.066	0.026	0.013	1.014 - 1.121
Precipitation (1-week lag)	0.962	0.015	0.009	0.935 - 0.991
**Smooth terms**	**edf**	***χ2***	***P*-value**	
Days since treatment	4.641	43.947	<0.001	
Trap ID (random effect)	0.939	15.115	<0.001	
Calendar days (random effect)	0.847	5.495	0.011	

Psychodidae

**Model predictors**	**Coefficient**	**SE**	***P*-value**	**95% CI**
Temperature[1]	0.959	0.045	0.357	0.877 - 1.048
Precipitation (1-week lag)	0.954	0.028	0.090	0.904 - 1.007
**Smooth terms**	**edf**	***χ2***	***P*-value**	
Days since treatment	3.741	28.077	<0.001	
Trap ID (random effect)	0.851	5.370	0.012	
Calendar days (random effect)	0.678	1.986	0.086	

Model predictions are from individual GAMM models based on Equation 2 (see main text).
[1]Temperature was centred around the mean temperature. 95% CI: 95% confidence interval; edf: effective degrees of freedom; GAMM: Generalised additive mixed model; SE: standard error.

Table S2 Percent reduction in mosquito and non-target insect abundance across different VectoMax FG application frequencies

Taxon	Interval	Frequency	Reduction (%)	95% CI (%)	Difference (%)
Aedes albopictus	10-weeks	0.100	60.9	48.1 - 70.5	-
	9-weeks	0.111	64.7	51.8 - 74.2	3.8
	8-weeks	0.125	69.1	56.0 - 78.3	4.4
	7-weeks	0.143	73.9	60.9 - 82.6	4.8
	6-weeks	0.167	79.2	66.6 - 87.0	5.3
	5-weeks	0.200	84.7[1]	73.1 - 91.3	5.5
	4-weeks	0.250	90.4	80.6 - 95.3	5.7[2]
	3-weeks	0.333	95.6	88.8 - 98.3	5.2
	2-weeks	0.500	99.0	96.3 - 99.8	3.4
Culex spp.	10-weeks	0.100	75.8	64.1 - 83.7	-
	9-weeks	0.111	79.3	67.9 - 86.6	3.5
	8-weeks	0.125	83.0[1]	72.2 - 89.6	3.7
	7-weeks	0.143	86.8	76.9 - 92.5	3.8[2]
	6-weeks	0.167	90.6	81.9 - 95.2	3.8
	5-weeks	0.200	94.1	87.1 - 97.3	3.5
	4-weeks	0.250	97.1	92.3 - 98.9	3.0
	3-weeks	0.333	99.1	96.7 - 99.8	2.0
	2-weeks	0.500	99.9	99.4 - 100	0.8
Chironimidae	10-weeks	0.100	43.6	30.0 - 54.5	-
	9-weeks	0.111	47.0	32.6 - 58.3	3.4
	8-weeks	0.125	51.1	35.9 - 62.7	4.1
	7-weeks	0.143	55.9	39.9 - 67.6	4.8
	6-weeks	0.167	61.5	44.8 - 73.2	5.6
	5-weeks	0.200	68.1	50.9 - 79.3	6.6
	4-weeks	0.250	76.1	58.9 - 86.1	8.0
	3-weeks	0.333	85.1[1]	69.4 - 92.8	9.0
	2-weeks	0.500	94.3	83.1 - 98.1	9.2[2]
Psychodidae	10-weeks	0.100	55.5	30.1 - 71.6	-
	9-weeks	0.111	59.3	32.8 - 75.3	3.8
	8-weeks	0.125	63.6	36.1 - 79.3	4.3
	7-weeks	0.143	68.6	40.1 - 83.5	5.0
	6-weeks	0.167	74.1	45.0 - 87.8	5.5
	5-weeks	0.200	80.2[1]	51.1 - 92.0	6.1
	4-weeks	0.250	86.8	59.1 - 95.7	6.6[2]
	3-weeks	0.333	93.2	69.6 - 98.5	6.4
	2-weeks	0.500	98.2	83.3 - 99.8	5.0

Percent reductions and their 95% confidence intervals are based on predictions from Equation 3 (see main text). The difference indicates marginal gain by incrementally reducing the application interval by 1 week. [1]Interval where 'sufficient' suppression has been reached (i.e. >80%). [2]Interval with maximal marginal gain.

Table S3 Reduction in mosquito and non-target insect abundance as a function of VectoMax FG application frequency, temperature and precipitation

**Taxon**	**Parameter**	**Coefficient**	**95% CI**	***P*-value**
Aedes albopictus	Application frequency	0.000129	0.000002 - 0.007026	< 0.001
	Temperature	1.053997	0.978194 - 1.135675	0.167
	Precipitation	0.929824	0.851159 - 1.015759	0.107
Culex spp.	Application frequency	0.000001	0.000000 - 0.000036	< 0.001
	Temperature	1.205567	1.112064 - 1.306932	< 0.001
	Precipitation	0.917851	0.849206 - 0.992045	0.031
Chironomidae	Application frequency	0.003279	0.000378 - 0.028436	< 0.001
	Temperature	1.061529	1.002815 - 1.123680	0.034
	Precipitation	0.984610	0.954324 - 1.015657	0.330
Psychodidae	Application frequency	0.000307	0.000003 - 0.027928	< 0.001
	Temperature	0.854815	0.759647 - 0.961906	0.009
	Precipitation	0.963782	0.917066 - 1.012878	0.146

Coefficients and 95% confidence intervals (CIs) are derived from Equation 3 (see main text).
[1]Temperature was centred around the mean temperature. 95% CI: 95% confidence interval.
